# Assessing microbial diversity in soil samples along the Potomac River: implications for environmental health

**DOI:** 10.1128/spectrum.02540-23

**Published:** 2023-10-24

**Authors:** Alexandra Taraboletti, Alexus King, Yasheka Dixon, Oshane Orr, Chevell Parnell, Yasheika Watson, Bruce Nash, Chimdimnma Esimai, George Ude

**Affiliations:** 1 Chemistry Program, Division of Sciences and Mathematics, University of the District of Columbia, Washington, DC, USA; 2 Biology Program, Division of Sciences and Mathematics, University of the District of Columbia, Washington, DC, USA; 3 Cold Spring Harbor Laboratory, Cold Spring Harbor, New York, USA; 4 Department of Natural Sciences, Bowie State University, Bowie, Maryland, USA; University of Mississippi, Oxford, Mississippi, USA

**Keywords:** metagenomics, environmental microbiology, soil microbiology, Potomac River, undergraduate research

## Abstract

In this study, we investigated the microbial diversity and community composition of soil samples collected from various sites along the Potomac River within an urbanized region. The study integrates microbial analysis into an undergraduate chemistry curriculum, bridging theoretical education with practical, real-world applications. Our findings revealed the presence of both typical marine soil bacteria and bacterial taxa indicative of urbanization and waste runoff. Notably, the identified taxa shared among all samples demonstrated a strong presence of *Burkholderiaceae*, *Nitrosomonadaceae*, and *Pedosphaeraceae*, which are associated with agricultural pollution, organochlorine pesticide contamination, and bromochloromethane pollution. We observed significant variations in microbial community diversity across different sampling sites, emphasizing the influence of environmental factors on microbial abundance and diversity. These insights carry significant implications for understanding the consequences of urbanization on soil microbial communities along the Potomac River and can inform strategies for managing and preserving these ecosystems. Further research is warranted to elucidate the effects of soil health and microbial diversity in this region.

**IMPORTANCE:**

This study integrates microbial analysis into an undergraduate chemistry class, offering students a hands-on approach to environmental research. We examined the soil along the urbanized Potomac River, discovering a mix of common marine microbes and others that are indicators of urban waste and pollution. Our findings provide valuable insights into the environmental impacts of urbanization on soil health and reveal the effectiveness of using modern genetic tools to teach students about real-world issues. This innovative educational approach not only deepens students’ understanding of chemistry and ecology but also prepares them to be thoughtful, informed participants in addressing contemporary environmental challenges while shedding light on the state of the soil microbiome near and around the DC metro area.

## INTRODUCTION

The Potomac River, renowned for its rich historical significance, flows through the Mid-Atlantic region of the USA, traversing the Potomac Highlands before draining into the Chesapeake Bay. The river contains a drainage area of about 14,500 square miles and is approximately 405 miles long. The Potomac basin stretches across parts of four states (Maryland, Pennsylvania, Virginia, and West Virginia) as well as the District of Columbia. The Potomac basin is the second largest watershed in the Chesapeake Bay watershed (1). This includes the land areas where water drains toward the mouth of the Potomac, such as the Anacostia River (8.5 miles long), which empties into the Potomac River at Hains Point in Washington, DC (2, 3). The watershed contains a large population, mostly located in the Washington metropolitan area, with forest being the largest land use and agriculture and urban areas being the second largest land uses in the upper and lower basins, respectively.

Human population growth, industrialization, and urbanization have caused a drastic increase in pollution levels within the Potomac basin (4 – 10). Washington, DC, has a long history of water pollution, with the Potomac and Anacostia rivers subjected to chemical pollution for over 200 years. The Anacostia River, in particular, is one of the top 10 most polluted rivers in the USA, containing sewage, metals, polycyclic aromatic hydrocarbons (PAHs), and polychlorinated biphenyls (PCBs) (9). Excessive nutrient inputs (mainly nitrogen from nonpoint sources) have caused the eutrophication of surface waters (11). The Potomac River is also plagued by high bacterial growth due to sewage runoff and improper waste disposal, leading to the river being used as a sewage drain (10). These poor waste management practices and the resulting bacterial growth have led to the formation of cyanobacteria blooms in years of drought and low river volume. The blooms deplete oxygen levels (hypoxic bottom-water dissolved oxygen) and result in the rivers being considered unswimmable and unfishable (7, 10, 12), as confirmed by the Potomac Conservancy (2020), which gave the river a grade of B (13). These human-influenced increases in waste and nutrient loads (including discharge from sewage treatment plants, atmospheric deposition, and urban/agricultural runoff) have all negatively impacted the Potomac basin.

In response to these issues, recent initiatives have been launched to maintain the cleanliness and health of the Potomac River, including the Clean Rivers Project, which aims to reduce combined sewer overflows and increase community monitoring of pollutants and toxins (14). Sewer separation is just one component of the plan to mitigate combined sewer overflows to the Potomac River and is part of the larger project to clean all three waterways in the District. There has also been a heightened level of community monitoring of pollutants and toxins in the Potomac River, particularly through the efforts of the Interstate Commission on the Potomac River Basin (ICPRB). This increased scrutiny has provided valuable insights into the health of the river and helped to further galvanize support for the protection and preservation of this important waterway.

In line with these efforts, this study aims to investigate the current environmental health of the Potomac River region in the DC metro area via a PCR amplicon study of soil samples from various locations along the basin. Soil hosts a wide range of microorganisms that play crucial roles in the ecosystem, and this is especially true for freshwater ecosystems like the Potomac River. The microbial communities present in soil have been shown to be a marker of and have a significant impact on the overall health of these ecosystems. By examining the soil samples collected along the Potomac River, we can gain valuable insights into the connection between soil microbes and the health of the river basin. The findings of this research have the potential to inform management practices aimed at maintaining and improving the health of the Potomac River for future generations.

## MATERIALS AND METHODS

### Sample collection

The river soil samples were collected in 50-mL sterile conical tubes, in triplicate, at a distance of 3–5 m from the banks of the Potomac River and a 6-inch depth from the soil surface. Ethanol and paper towels were used interchangeably between each soil sample collection to ensure clean, sterile tools. Samples were transported to the laboratory within an hour of collection and stored in a −20°C refrigerator until further processed.

### Physiochemical measurements

Relative nitrogen, phosphorus, and potassium levels were measured using a LaMotte NPK Soil Test Kit. Approximately 0.5 g of soil was extracted for each sample, and the nitrogen, phosphorus, and potassium levels were recorded as specified in the kit procedure. The pH of each sample was collected using a Soil Condition Meter. The soil meter probe was inserted directly into the soil sample and allowed to equilibrate for 1 min prior to recording the sample pH.

### DNA extraction

The tubes were ultrasonicated for 1 min each to achieve cell disruption. Extraction was completed using a Qiagen DNeasy PowerSoil Kit protocol (8). Approximately 0.25 g of each soil sample was weighed and recorded. To achieve cell disruption, the samples were ultrasonicated for 1 min each. Samples were then stored at −20°C until PCR was performed. NanoDrop was used to confirm the quality and concentration of the DNA obtained from the soil samples (Table S1).

### PCR/gel electrophoresis

DNA products were PCR amplified using primers “515F–806R” targeting the V4 region of the 16S SSU rRNA—used by the Earth Microbiome Project (9). Primer sequences are as follows: 515F (Parada)–806R (Apprill), F: GTGYCAGCMGCCGCGGTAA; R: GGACTACNVGGGTWTCTAAT. Primers and primer constructs were designed by Greg Caporaso (10, 11). Modifications to primer degeneracy were done by the labs of Jed Furhman (12) and Amy Apprill (13). Forward-barcoded constructs were redesigned by Walters (14). Amplification conditions were performed in a 25-µL reaction volume and consisted of 13 µL of nuclease-free water, 10 µL of 2× PCR master mix, 0.5 µL forward primer, 0.5 µL reverse primer, and 1 µL soil DNA. The thermal cycle was programmed for 120 s at 94°C for initial denaturation, followed by 35 cycles of 45 s at 94°C for denaturation, 60 s at 50°C for annealing, 90 s at 72°C for extension, and a final extension at 72°C for 10 min. PCR products were examined by gel electrophoresis at 100 V for 45 min in a 1% (w/v) agarose gel with ethidium bromide in 1× TAE buffer and compared to a 1-kb DNA ladder (Fig. S1).

### 16S rRNA amplicon sequencing and analysis

After demultiplexing to assign reads to samples at the New York Genome Center, the resulting FASTQ files were placed in the CyVerse Discovery Environment so that sequences could be analyzed in the DNA Subway Purple Line (15, 16), which is a graphic user interface for QIIME2. Using the demultiplexed sequence counts summary (Fig. S2), low-quality sequences were trimmed to position 243 (TruncLenF: 243, TruncLenR: 243). After trimming, samples were rarified (rarification depth Min: 1, Max: 5,000), with sampling depths based on the frequency per sample data (Fig. S3). Operational taxonomy unit (OTU) tables were then generated by matching to the Silva (16S/18S rRNA) database.

### Statistics and analysis

The Marker Data Profiling (MDP) module in MicrobiomeAnalyst (17, 18) was used to further process the OTU tables generated by DNA Subway. An overview of the library size revealed two sample outliers with <10 read counts (YD1 and CP3; Fig. S4). These samples were removed from further grouped analyses. To remove low-quality or uninformative features, data filtering was done using a low count filter (minimum count = 4, 20% sample prevalence cutoff) and a low variance filter (10% removed based on inter-quantile range). To deal with the variations in sample depth and the sparsity of the data, normalization (total sum scaling) was applied. Using this data set, alpha diversity, beta diversity, pie chart, dendrogram, and abundance bar graphs were all generated.

## RESULTS

### Sample collection and study site characteristics

**Fig 1:**
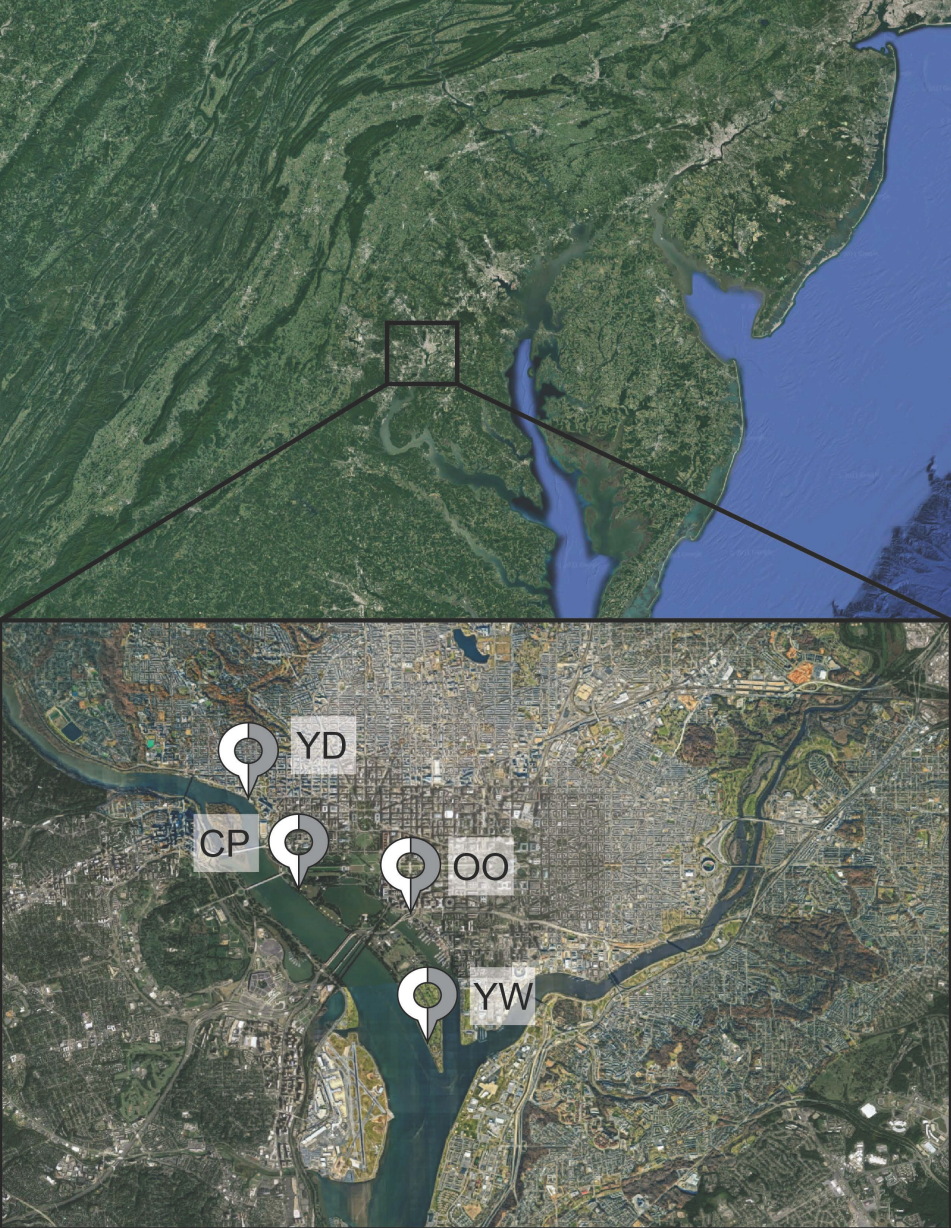
Sample site map. Map of the Potomac watershed, with the DC metro area highlighted. Pins with site names (YD, CP, OO, and YW) show where the Potomac River samples were collected. Map data ©2023 Google.

Soil samples were collected in September 2021 from four locations/sites within the Potomac watershed (Table 1). The four sites, namely, OO, YD, YW, and CP, are each located at different points along the Potomac River, within the boundary of Washington, DC (Fig. 1). Sampling was performed during a period in which the average temperature was 73°F and the average rainfall was 0.3 inches (https://www.weather.gov/; Fig. S5). In this period, the season was characterized as dry; however, for the 2 days between the collection of the samples, there was light rain in the region.

**TABLE 1:** Soil sampling sites along the Potomac River

SN#	Date/time	Site of collection	GPS coordinates
OO1	2:30 p.m. 09/22/2021	SE DC Wharf waterside	Latitude: 38.8821 Longitude: −77.0281
OO2	2:35 p.m. 09/22/2021	SE DC Wharf waterside	Latitude: 38.8821 Longitude: −77.0281
OO3	2:40 p.m. 09/22/2021	SE DC Wharf waterside	Latitude: 38.8821 Longitude: −77.0281
YD1	2:20 p.m. 09/20/2021	Georgetown Waterfront Park	Latitude: 38.900559 Longitude: −77.058686
YD2	2:26 p.m. 09/20/2021	Georgetown Waterfront Park	Latitude: 38.900559 Longitude: −77.058686
YD3	2:37 p.m. 09/20/2021	Georgetown Waterfront Park	Latitude: 38.900559 Longitude: −77.058686
YW1	7:30 p.m. 09/30/2021	1138–1140 Ohio Dr. SW	Latitude: 38.87792 Longitude: −77.03797
YW2	7:32 p.m. 09/30/2021	1138–1140 Ohio Dr. SW	Latitude: 38.87795 Longitude: −77.03794
YW3	7:32 p.m. 09/30/2021	1138–1140 Ohio Dr. SW	Latitude: 38.87795 Longitude: −77.03794
CP1	2:34 p.m. 09/22/2021	Tidal Basin Washington DC	Latitude: 38.885904 Longitude: −77.049705
CP2	2:35 p.m. 09/22/2021	Tidal Basin Washington DC	Latitude: 38.885904 Longitude: −77.049705
CP3	2:36 p.m. 09/22/2021	Tidal Basin Washington DC	Latitude: 38.885904 Longitude: −77.049705

These sites were chosen as they lay along a portion of the river still heavily impacted by combined sewer overflows (CSOs) (19 – 21). Washington, DC, has been making progress in cleaning up the Anacostia and Potomac rivers through the court-mandated project known as Clean Rivers (21). The project consists of 18 miles of underground tunnels designed to capture CSO before it reaches the rivers. One important tunnel, the “Potomac River Tunnel,” has still yet to be constructed, leaving much of the DC river region—namely, the sampled areas—polluted (19).

### Soil physiochemical measurements

The physiochemical measurements (Table 2) of the soil samples collected along the Potomac River revealed that relative nitrogen levels were 20 ppm or below in all samples. The relative phosphorus levels ranged from 4 to 10 ppm, while all potassium levels were 80 ppm or above. In addition, the pH levels of the soil samples collected along the Potomac River ranged from pH 6.8 to 8.4, with the majority of samples being neutral to slightly basic.

**TABLE 2:** Soil physiochemical measurements

SN#	Sample mass	Nitrogen	Phosphorus	Potassium	pH
OO1	0.501 g	≤20 ppm	≤4 ppm	≥80 ppm	7.4
OO2	0.500 g	≤20 ppm	6 ppm	≥80 ppm	7.8
OO3	0.501 g	≤20 ppm	≤4 ppm	≥80 ppm	8.3
YD1	0.502 g	≤20 ppm	10 ppm	≥80 ppm	8.4
YD2	0.500 g	≤20 ppm	10 ppm	≥80 ppm	7.7
YD3	0.500 g	≤20 ppm	10 ppm	≥80 ppm	6.8
YW1	0.501 g	≤20 ppm	6 ppm	≥80 ppm	7.8
YW2	0.500 g	≤20 ppm	10 ppm	≥80 ppm	7.3
YW3	0.501 g	≤20 ppm	6 ppm	≥80 ppm	7.3
CP1	0.500 g	≤20 ppm	≤4 ppm	≥80 ppm	7.7
CP2	0.500 g	≤20 ppm	≤4 ppm	≥80 ppm	8.4
CP3	0.501 g	≤20 ppm	≤4 ppm	≥80 ppm	8.1

### DNA quality and amplification

DNA samples obtained in the extraction protocol were of good yield (>20 ng/µL) and quality (260/280 value >1.8). The measurements of isolated DNA, for quality and quantity, are presented in Table S1. Expected amplification was obtained in almost all samples—exhibiting strong bands as visualized with an agarose gel (Fig. S1). This suggests some error in the processing of samples YD1 and CP3, and they should be distinguished as outliers.

### Microbial diversity and community profiling

The amplified DNA from these four sites was analyzed via Illumina HiSeq paired-end sequencing, generating 29,164 clean reads (average 2,430 per sample; range from 1,426 to 4,367) and 666 OTUs (defined at 97% sequence similarity). More detailed summary statistics of the sequences can be found in the supplemental material. Samples YD3 and CP3, which had reads of 10 and 0, respectively, match samples found not amplified via PCR (Fig. S1 and S2) and were removed as outliers from further data analysis.

**Fig 2:**
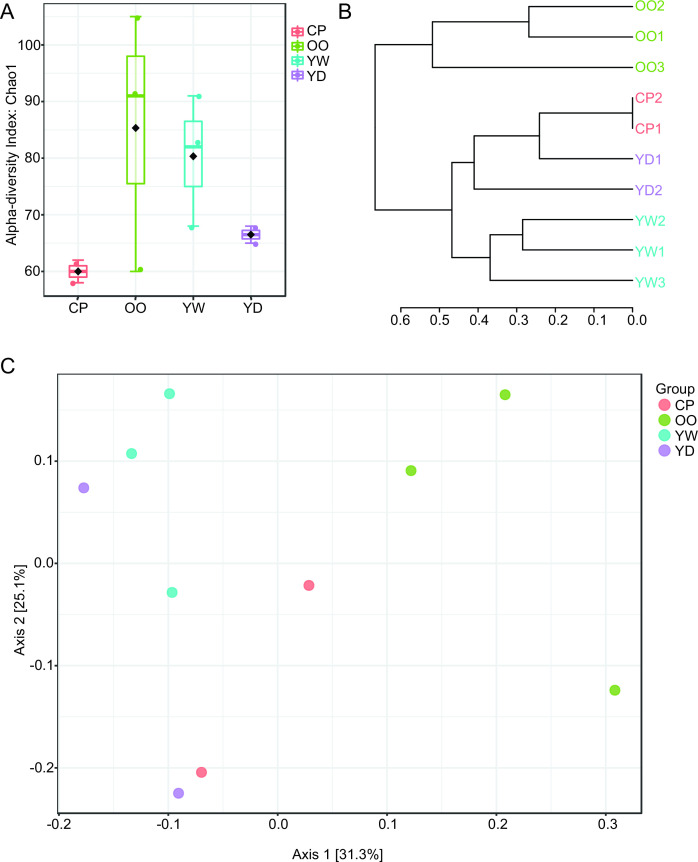
Microbial diversity. (**A**) Alpha diversity profiling of all samples, utilizing Chao1 diversity measures with the ANOVA statistical method. (**B**) Dendrogram analysis of all samples utilizing Bray-Curtis index distance measures with Ward clustering. (**C**) Beta diversity profiling of all samples utilizing the PCoA 2D ordination plot, with Bray-Curtis index distance measures and the PERMANOVA statistical method. Samples are grouped by the sampling site: CP (*n* = 2), OO (*n* = 3), YW (*n* = 3), and YD (*n* = 2).

The distribution of microbial alpha diversity indices is visualized in Fig. 2A. Overall, the mean values in alpha diversity indices varied among samples grouped by collection site along the river; however, these differences were not statistically significant (ANOVA, *P* > 0.05). Notably, communities sampled from a small tidal channel parallel to the main river body (DC Wharf; OO) showed higher mean alpha diversity when compared to all other samples.

Principal coordinates analysis [PCoA, =multidimensional scaling (MDS)] of the normalized OTUs based on Bray-Curtis distances (Fig. 2B) also showed a separation between soil samples collected along the main river body (CP, YD, and YW) versus those collected along the parallel channel (OO); this observation is reinforced through dendrogram analysis (Bray-Curtis distances, Ward clustering; Fig. 2C).

**Fig 3:**
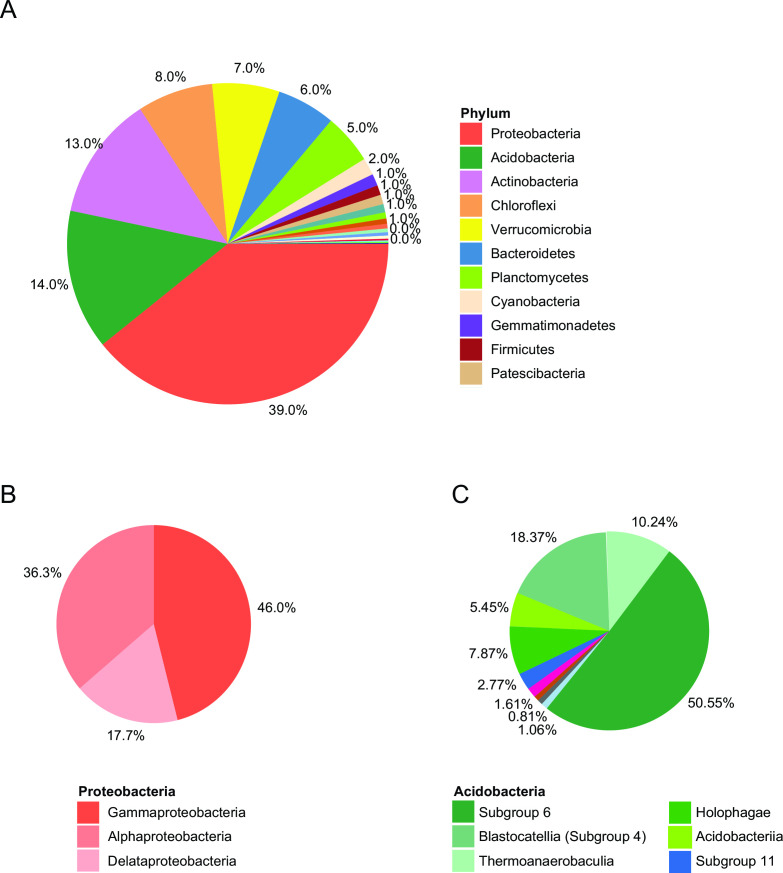
Microbial community profiling. (**A**) Pie chart representing the percentage of features found across all samples (*n* = 10) at the phylum level. Pie charts of the class-level composition of the (**B**) *Proteobacteria* phylum and the (**C**) *Acidobacteria* phylum.

Among the identified OTUs, members of bacteria were predominant (98.05%), and a small number of OTUs were classified in the domain of archaea (1.95%). Identification of the OTUs at finer taxonomic levels yielded 73 phyla, 167 classes, 417 orders, 705 families, 1,439 genera, and 570 species. At the phylum level, *Proteobacteria* was dominant (39.0%), followed by *Acidobacteria* (14.0%), *Actinobacteria* (14.0%), *Chloroflexi* (8.0%), *Verrucomicrobia* (7.0%), *Bacteroidetes* (6.0%), *Planctomycetes* (5.0%), *Cyanobacteria* (2.0%), *Gemmatimonadetes* (1.0%), and *Firmicutes* (1.0%); these top 10 bacterial phyla constituted 96% of the total OTUs (Fig. 3A). Among *Proteobacteria*, the class *Gammaproteobacteria* was predominant (47.5%), followed by *Alphaproteobacteria* (38.3%), and *Deltaproteobacteria* (14.2%; Fig. 3B); among *Acidobacteria*, subgroup 6 predominates (48.74%), with a noticeable presence of subgroup 4 (18.75%; Fig. 3C).

### Microbial composition analysis

**Fig 4:**
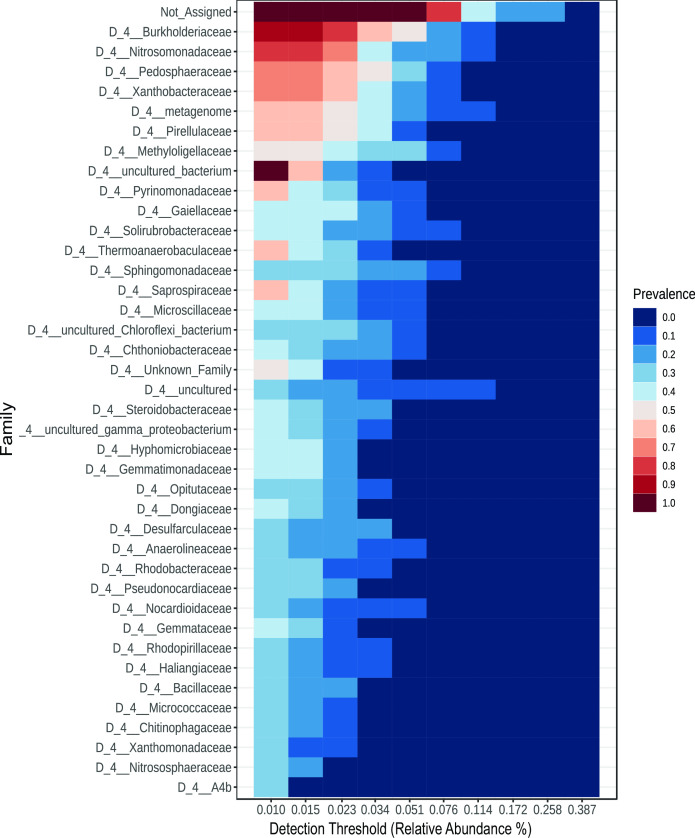
Microbial composition and taxon set analysis at the family level. Core microbiome threshold map of the 100 most abundant family-level taxa found across all of the sampling sites (*n* = 10).

Core microbiome threshold analysis at the family level revealed the top shared/identified taxa to be *Burkholderiaceae*, *Nitrosomonadaceae*, *Pedosphaeraceae*, *Xanthobacteraceae*, *metagenome, Pirellulaceae*, *Methyloligellaceae*, *Pyrinomonadaceae*, *Gaiellaceae*, and *Solirubacteraceae* (Fig. 4).

### Site-specific distribution of microbial composition

**Fig 5:**
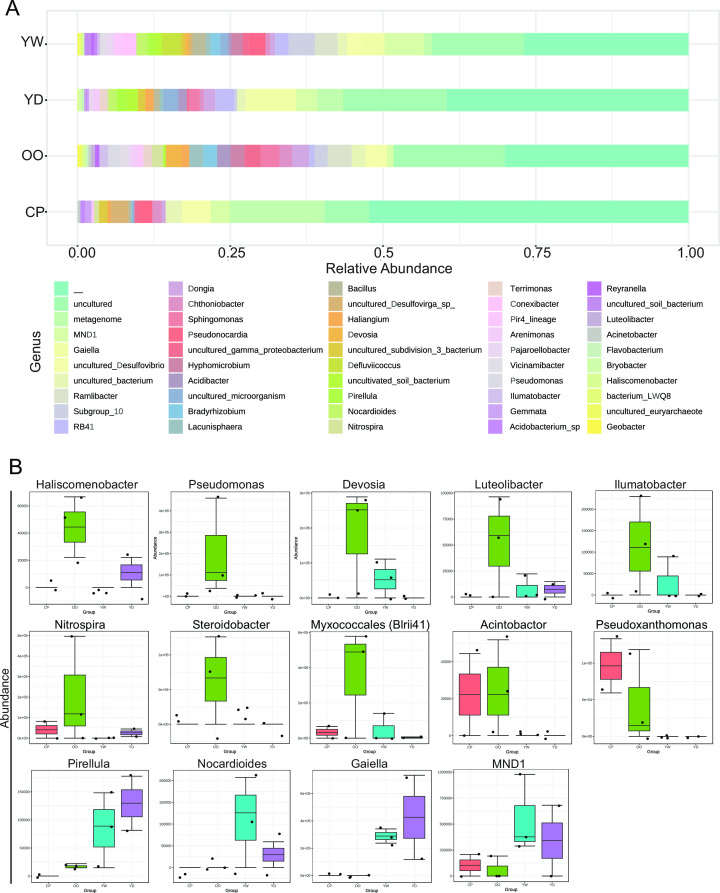
Site-specific distribution of microbial composition at the genus level. (**A**) Stacked bar plot showing the relative abundance of the top 50 taxa (genus level) found within sample groups. Samples are pooled by the site collection group. (**B**) Box plots highlighting specific abundance differences in taxa (genus level). Samples are pooled by the site collection group. CP (*n* = 2), OO (*n* = 3), YW (*n* = 3), and YD (*n* = 2). *P* < 0.05.

Grouped percent abundance data (Fig. 5A) highlights taxon abundance differences (genus level) based on the sampling site. Detailed box plot comparisons (Fig. 5B) show that *Haliscomenobacter*, *Pseudomonas*, *Devosia*, *Luteolibacter*, *Ilumatobacter*, *Nitrospira*, *Steriodobacter*, and *Myxococcales (Blrii41*) were found in higher abundance in location OO compared to the other sites. *Acinetobacter* and *Pseudoxanthomonas* were also found in higher abundance, together at sites OO and CP. *Pirellula*, *Nocardioides, Gaiella*, and *MND1* were, instead, found in high abundance in sites along the upper stretch of the river (YW and YD). All correlation tables can be found in Table S2.

## DISCUSSION

The present study reports physiochemical and bacterial information at the phylum and subgroup levels of soil collected along the DC metro region of the Potomac River as a means to relay information pertaining to the environmental health of the river basin.

Though soil nitrogen, phosphorous, and potassium (NPK) levels can vary depending on the specific environmental context and the types of plants or organisms present, all measured samples had relatively low nitrogen levels, low-medium phosphorous levels, and high potassium levels. Soil samples with low nitrogen levels, ranging from 20 ppm or below, and medium to low levels of phosphorus, ranging from 4 to 10 ppm, signal limited plant growth and productivity, which could lead to a decrease in overall biodiversity (15, 16). High levels of potassium, above 80 ppm, can indicate that excess fertilizer or manure application has occurred, leading to eutrophication in nearby waterways (17 – 19).

The group patterns of the soil microbial data from all samples (Fig. 2) displayed variation in alpha diversity indices among samples but without significant differences (Fig. 2A). However, the DC Wharf site samples showed higher mean alpha diversity, indicating potential variations in microbial community composition between sites. The PCoA and dendrogram analyses (Fig. 2B and C) revealed separation between samples collected along the main river body versus those collected along a small tidal channel, indicating possible discrepancies in human impact, water retention, and tidal/current effects. These patterns also corresponded to differences in soil phosphorous levels, with the samples collected along the tidal channel having slightly lower levels of phosphorous than those collected from the main river (Table 2).

The top taxa shared among all sampling sites match typical 16S rRNA-based analyses of phylum diversity found in soil, marine, and wastewater samples (Fig. 3) (22, 23). In particular, *Proteobacteria, Actinobacteria*, and *Acidobacterium* are well represented and often account for 90% of cultivated soil bacteria (Fig. 3A) (22)*. Proteobacteria* is a diverse group of bacteria that is found in a variety of aquatic and terrestrial environments. This phylum is a major contributor to the microbial communities in the Potomac River basin and is likely responsible for the cycling of organic matter, nitrogen, and other essential elements. *Actinobacteria* and *Acidobacterium* are a group of bacteria that are typically associated with soil and are thought to play an important role in the decomposition of organic matter nutrient cycling processes. *Proteobacteria* (gamma), *Actinobacteria*, and *Acidobacterium* (subgroup 6) (Fig. 3B and C) have been reported to thrive in soil with low levels of nitrogen (20, 21). These findings are consistent with the physicochemical results obtained from the soil samples collected along the Potomac River (Table 2), which showed low levels of nitrogen across all sites. The abundance of these bacterial groups in the soil samples may be attributed to their ability to utilize alternative sources of nitrogen, such as organic matter, or to their capacity to fix atmospheric nitrogen (20). The presence of these bacteria is expected, but it confirms that the soil is impacted by human activities (22, 23).

The significant presence of the phylum *Chloroflexi* in the samples may also indicate a shift in the environmental health of the river (Fig. 3A). Certain *Chloroflexi* bacteria are associated with halophilic and thermophilic environments, and their presence could suggest that the Potomac River basin is facing increased hydrological stress. *Chloroflexi* are known to be involved in organohalide respiration and have potential roles in the bioremediation of chlorinated compounds (24)—noted due to historic PCB pollution in the Chesapeake Bay region (25 – 28). *Chloroflexi* also play an important role in activated sludge water treatment plants (29, 30), and the presence of these bacteria may also be indicative of changes in the river basin’s nutrient cycle. *Chloroflexi* are known for their ability to break down organic matter, and their presence suggests that there may be an increase in the amount of organic matter entering the river basin.

The identified taxa (family level; Fig. 4) shared among all samples note the strong presence of *Burkholderiaceae*, *Nitrosomonadaceae,* and *Pedosphaeraceae*. These taxa are indicative of nitrogen cycling and an environment with by-products of sewage and agricultural runoff (24, 25). *Burkholderiaceae* has been positively correlated with aerobic chemoheterotrophy, aromatic compound degradation, and ureolysis. *Xanthobacteraceae* and *Methyloligellaceae* are typically found in environments with high levels of carbon, which could be due to the abundance of urban settings present along the Potomac River basin (26 – 28). *Pirellulaceae*, *Pyrinomonadaceae*, *Sphingomonadaceae*, and *Saprospiraceae* are known to be associated with soil, marine sediments, and biofilms (29, 30 *Sphingomonadaceae*, *Solibacteraceae*, and *Nitrosomonadaceae* have all been positively correlated with aerobic nitrite oxidation, aerobic ammonia oxidation, and nitrification (all *P* < 0.05) (31). The abundance of these bacterial taxa is possibly an indication of elevated levels of urbanization and industrial activity in the vicinity. Taxon set analysis (Fig. S6), which compares environmental taxon sets to the shared family-level taxa, suggests that these top taxa are strongly correlated with agricultural pollution, organochlorine pesticide contamination, and bromochloromethane pollution.

Though many features found in all of the samples were associated with urban settings, Fig. 5 notes differences in taxon abundance found based on sampling sites, and these differences correspond to environmental differences as supported by multivariate analyses (Fig. S7). For instance, genus-level taxa, such as *Acinetobacter* and *Pseudoxanthomonas,* were more abundant in samples from sites OO and CP, whereas *Pirellula*, *Nocardioides*, *Gaiella*, and *MND1* were more abundant in samples from sites YW and YD. *Haliscomenobacter*, *Pseudomonas*, *Devosia*, *Luteolibacter*, *Ilumatobacter*, *Nitrospira*, *Steriodobacter*, and *Myxococcales (Blrii41*) were more abundant in samples from site OO. Taxa highly represented from sites YW and YD play a central role in carbon cycling through methane and plant and algal degradation, while taxa from sites OO and CP are involved in complex carbon utilization and activated sludge sites. While our sampling was primarily focused on identifying dominant microbial species in the region, we recognize that the scale and design of our sampling might not have captured the full range of community patchiness. Future endeavors in this region could benefit from a more intensive sampling strategy, specifically targeting the nuances of community composition patchiness driven by both natural and anthropogenic factors.

### Conclusion

This research provides valuable insights into the microbial diversity and community composition of soil samples collected from various locations along the urbanized stretch of the Potomac River. Our findings underscore the notable variations in microbial community structure and diversity across different sampling sites, emphasizing the influence of environmental factors on microbial abundance. We identified specific bacterial taxa associated with high levels of urbanization, waste sites, and agricultural pollution. Additionally, the study brings attention to potential disparities in human impact among the soil samples. These contribute to a better understanding of the complex interplay between urbanization and soil microbial communities along the Potomac River. Further research is warranted to more comprehensively explore the impacts of soil health and microbial diversity in this region, with the aim of informing effective strategies for maintaining and improving the health of this vital waterway for future generations.

## Data Availability

All metagenomic data are publicly available in the ArrayExpress database under accession no. E-MTAB-13365.

## SUPPLEMENTAL MATERIAL

Supplemental material spectrum.02540-23-s0001.docx Tables S1 and S2; Fig. S1 to S7.

ASM does not own the copyrights to Supplemental Material that may be linked to, or accessed through, an article. The authors have granted ASM a non-exclusive, world-wide license to publish the Supplemental Material files. Please contact the corresponding author directly for reuse.
