# Supplementary material for: Assessing microbial diversity in soil samples along the Potomac River: implications for environmental health
Source: Microbiol Spectr. 2023 Oct 24;11(6):e02540-23. doi: 10.1128/spectrum.02540-23 (PMC10714937; doi:10.1128/spectrum.02540-23)
Supplement: Supplemental material — Tables S1 and S2; Fig. S1 to S7. [file spectrum.02540-23-s0001.docx]

# Supplementary Information

**Supplementary Table 1: DNA Concentrations**

| SN# | Sample Mass (g) | DNA Conc. (ng/uL) | DNA 260/280 | DNA 260/230 |
| --- | --- | --- | --- | --- |
| OO1 | 0.2530 | 47.2 | 1.94 | 1.28 |
| OO2 | 0.2590 | 90.1 | 1.92 | 1.99 |
| OO3 | 0.2521 | 40.1 | 1.91 | 2.53 |
| YD1 | 0.2075 | 15.5 | 1.92 | 1.49 |
| YD2 | 0.2226 | 23.4 | 2.01 | 1.89 |
| YD3 | 0.2438 | 52.0 | 1.91 | 1.55 |
| YW1 | 0.2530 | 30.2 | 1.90 | 1.91 |
| YW2 | 0.2521 | 20.0 | 1.95 | 2.06 |
| YW3 | 0.2534 | 37.7 | 1.95 | 1.79 |
| CP1 | 0.2620 | 41.6 | 1.96 | 1.90 |
| CP2 | 0.2690 | 37.6 | 1.97 | 1.75 |
| CP3 | 0.2600 | 43.1 | 1.98 | 1.78 |

**Supplementary Figure 1: PCR Product Gel**


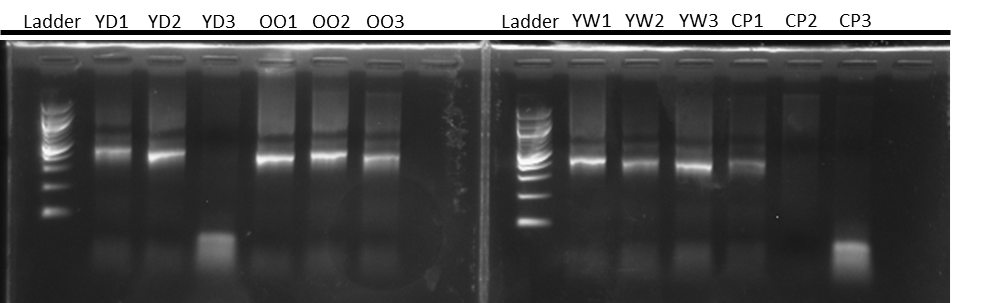


**Supplementary Figure 2**

# **Demultiplexed sequence counts summary**

| Minimum: | 227 |
| --- | --- |
| Median: | 16659.0 |
| Mean: | 13433.666666666666 |
| Maximum: | 24542 |
| Total: | 161204 |

Demultiplexed sequence length summary

Forward Reads

|  |  |
| --- | --- |
| **Total Sequences Sampled** | 10000 |
| **2%** | 251 nts |
| **9%** | 251 nts |
| **25%** | 251 nts |
| **50% (Median)** | 251 nts |
| **75%** | 251 nts |
| **91%** | 251 nts |
| **98%** | 251 nts |

Reverse Reads

|  |  |
| --- | --- |
| **Total Sequences Sampled** | 10000 |
| **2%** | 251 nts |
| **9%** | 251 nts |
| **25%** | 251 nts |
| **50% (Median)** | 251 nts |
| **75%** | 251 nts |
| **91%** | 251 nts |
| **98%** | 251 nts |

**Supplementary Figure 3: Alpha Rarefaction**


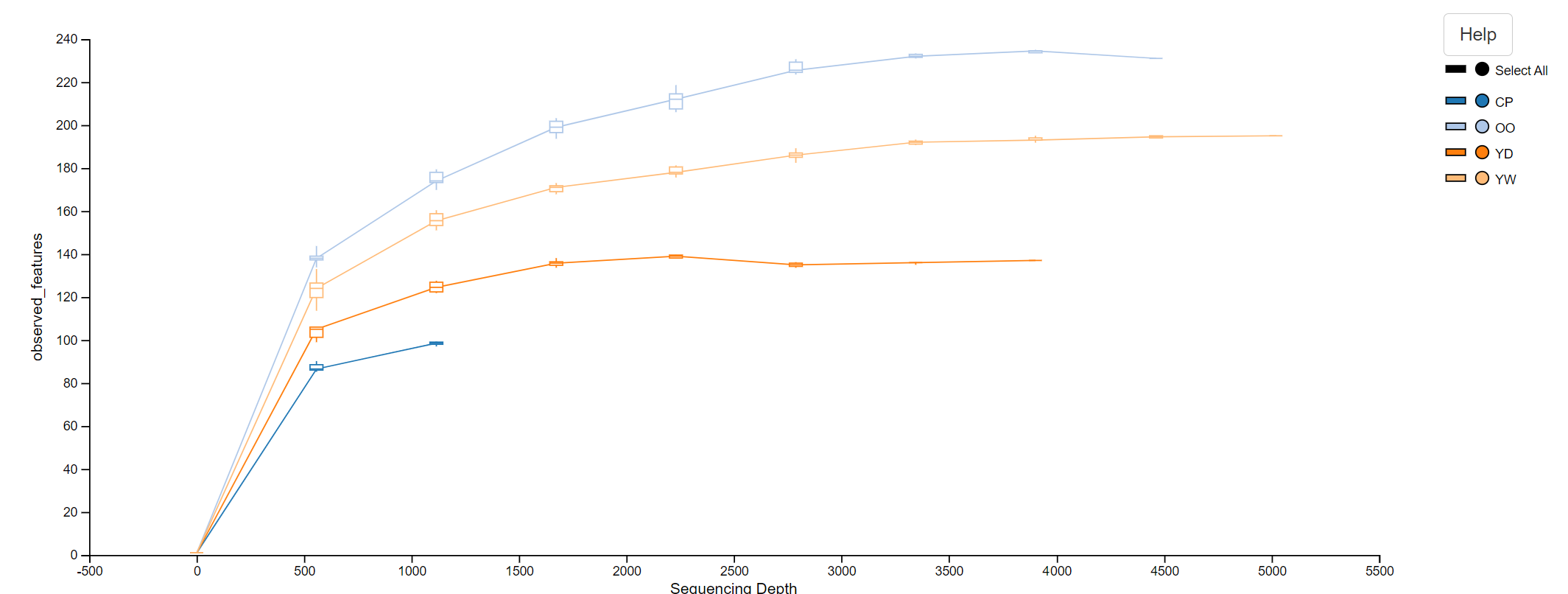


**Supplementary Figure 4: Library size**


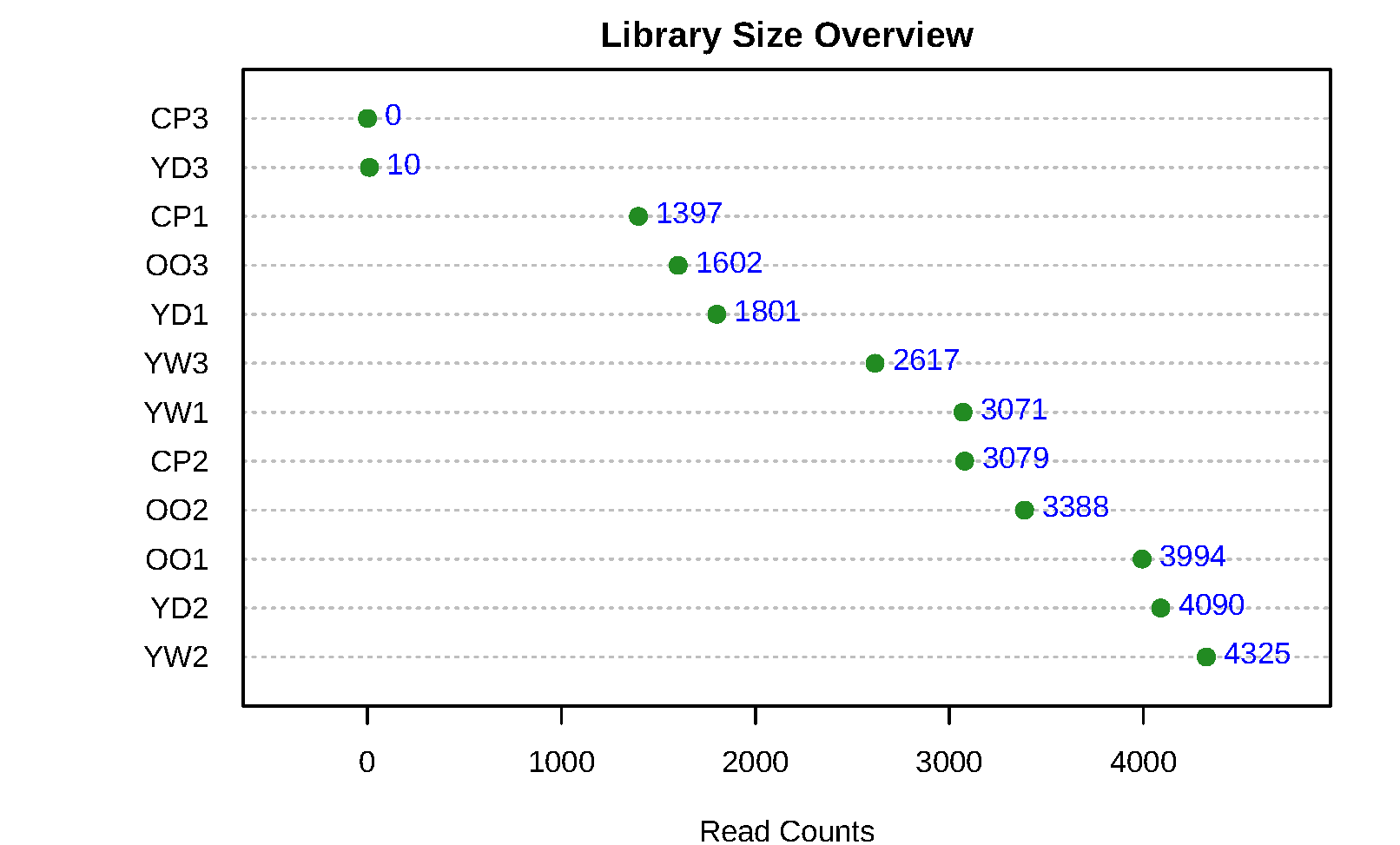


**Supplementary Figure 5: Weather Data**


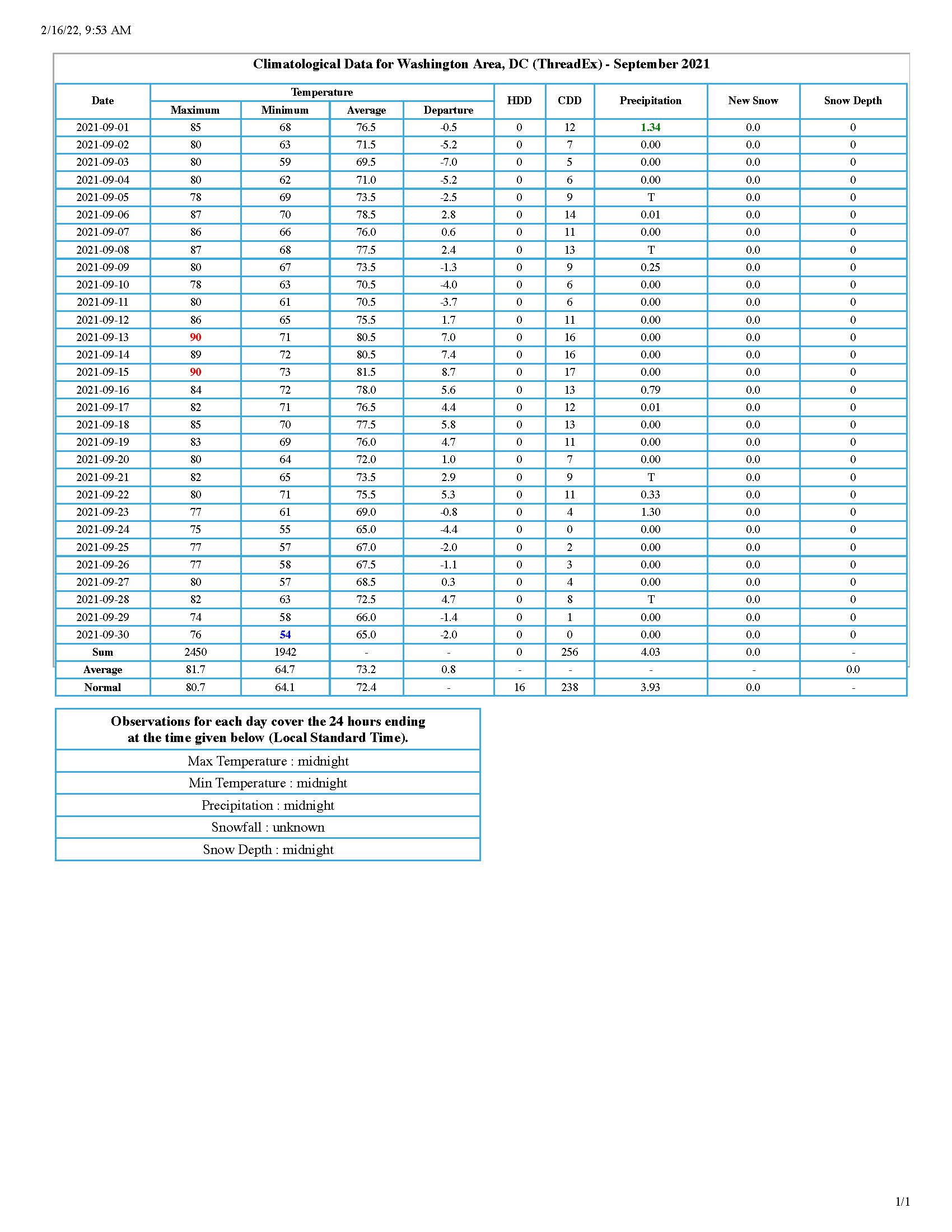


Supplementary Figure 6: Taxon Set Analysis Table


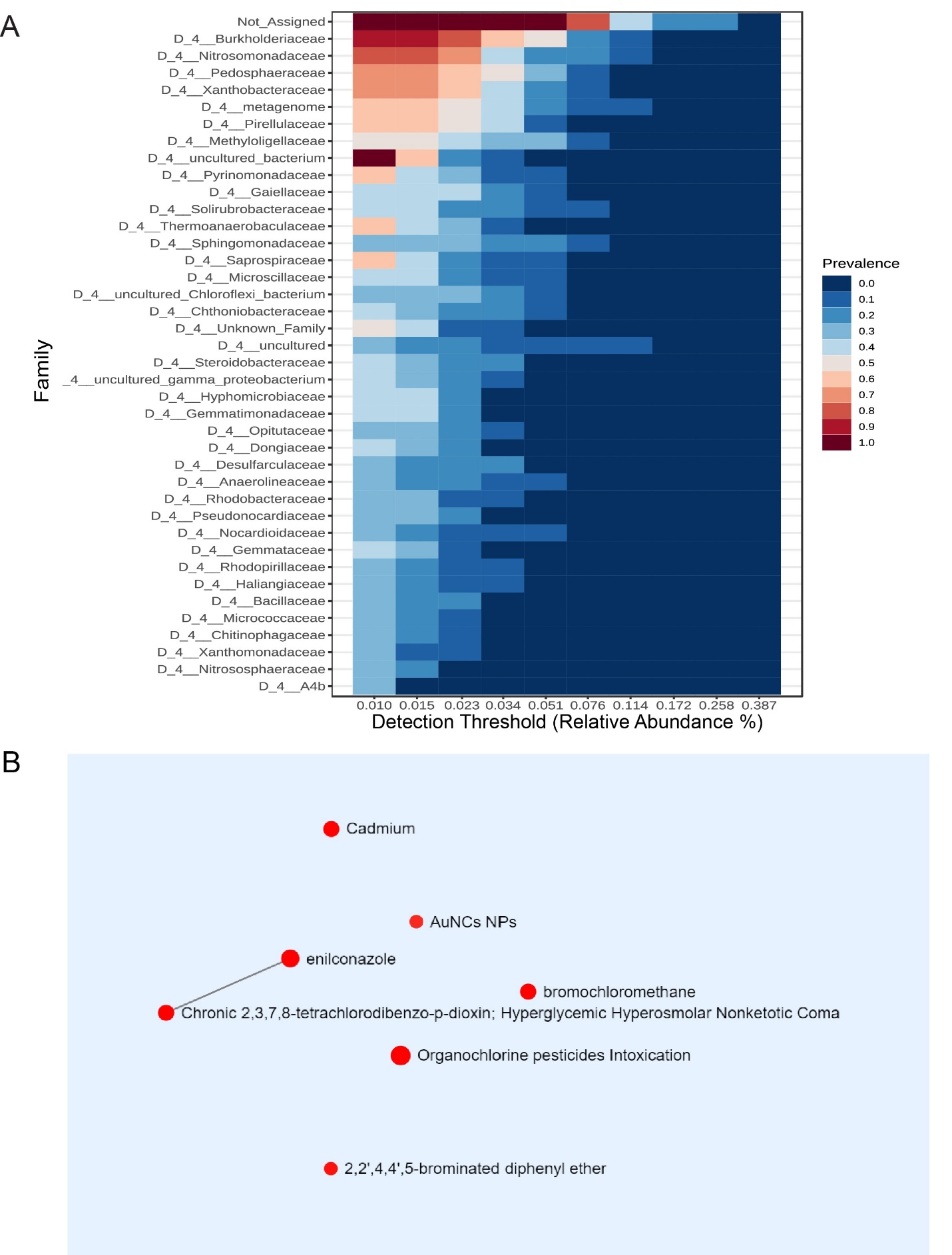


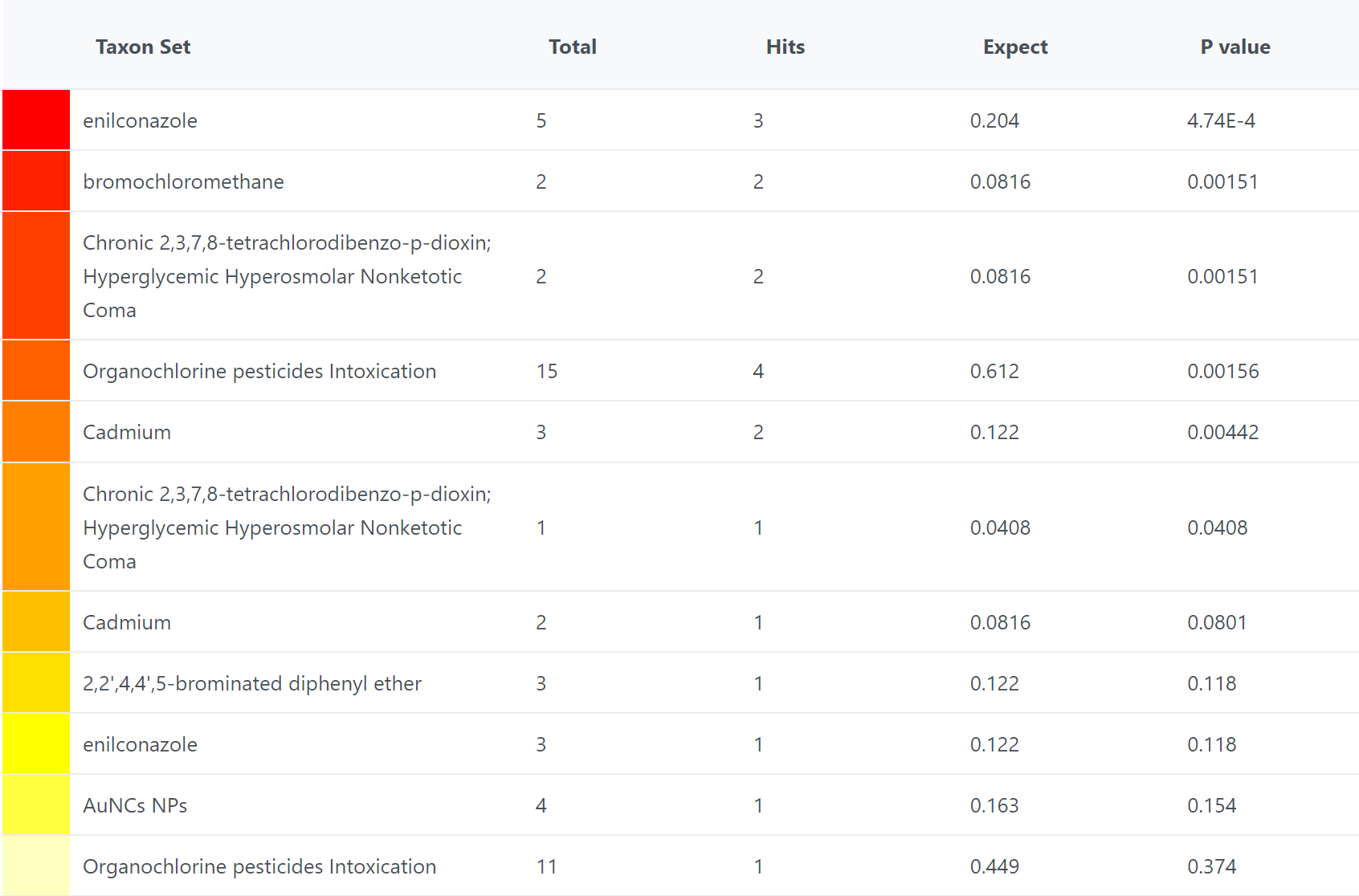


Taxon set analysis, enrichment analysis of the top family level taxa found in all samples compared to known environmental taxon set. Each node represents a taxon set, with its color based on its p-value and the size based on the number of matched hits. P<0.05

Supplementary Figure 7: Multivariate Analysis (with environmental features)


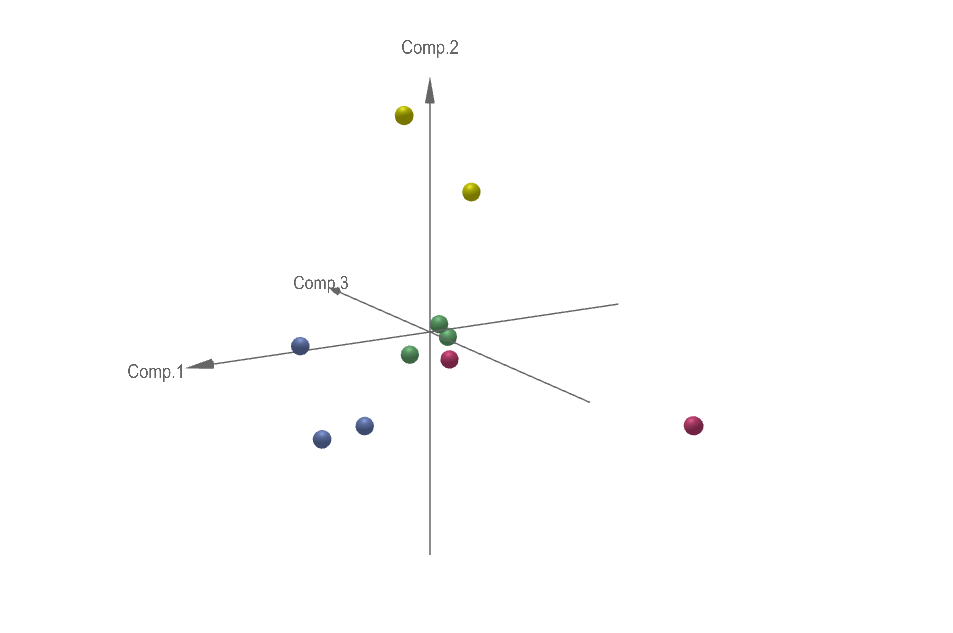


Multivariate profiling of all samples combining environmental data and microbial, and Multiple co-inertia analysis (MCIA) 3D ordination plot, with Pearson matrix method. Samples are colored by sampling site – CP (red, n=2), OO (green, n=3), YW (blue, n=3), and YD (yellow, n=2).

Supplementary Table 2: Correlation tables

|  | Correlation | T-Stat | P-Value | FDR |
| --- | --- | --- | --- | --- |
| D_5__Pirellula | 0.77856 | 3.509 | 0.007974 | 0.74267 |
| D_5__Gaiella | 0.73781 | 3.0916 | 0.014853 | 0.74267 |
| D_5__Pseudoxanthomonas | -0.69491 | -2.7333 | 0.025715 | 0.75641 |
| D_5__Chryseolinea | -0.58454 | -2.0377 | 0.075945 | 0.75641 |
| D_5__TG_45 | -0.5788 | -2.0075 | 0.079581 | 0.75641 |
| D_5__uncultured_bacterium | -0.55545 | -1.8893 | 0.095526 | 0.75641 |
| D_5__bacterium_WX65 | 0.53452 | 1.7889 | 0.11143 | 0.75641 |
| D_5__PAUC26f | 0.53434 | 1.788 | 0.11158 | 0.75641 |
| D_5__Acinetobacter | -0.5224 | -1.7328 | 0.12136 | 0.75641 |
| D_5__Mycobacterium | 0.51967 | 1.7204 | 0.12367 | 0.75641 |
| D_5__uncultivated_soil_bacterium_clone_C112 | 0.51383 | 1.6941 | 0.12871 | 0.75641 |
| D_5__uncultured_Chloroflexi_bacterium | 0.51288 | 1.6898 | 0.12953 | 0.75641 |
| D_5__Bryobacter | 0.50286 | 1.6455 | 0.13849 | 0.75641 |
| D_5__Haliangium | -0.49822 | -1.6253 | 0.14276 | 0.75641 |
| D_5__Streptomyces | 0.49323 | 1.6037 | 0.14744 | 0.75641 |
| D_5__Stenotrophobacter | -0.48795 | -1.5811 | 0.1525 | 0.75641 |
| D_5__uncultured_bacterium_5G4 | -0.47734 | -1.5365 | 0.16298 | 0.75641 |
| D_5__uncultured_microorganism | 0.47312 | 1.5189 | 0.16726 | 0.75641 |
| D_5__Solirubrobacter | 0.46697 | 1.4936 | 0.17362 | 0.75641 |
| D_5__Desulfobulbus | -0.46455 | -1.4838 | 0.17617 | 0.75641 |
| D_5__mle1_7 | 0.4591 | 1.4617 | 0.18197 | 0.75641 |
| D_5__Aquicella | 0.45733 | 1.4545 | 0.18388 | 0.75641 |
| D_5__Geobacter | -0.45594 | -1.449 | 0.18538 | 0.75641 |
| D_5__Candidatus_Udaeobacter | 0.45472 | 1.4441 | 0.18671 | 0.75641 |
| D_5__MND1 | 0.44714 | 1.4139 | 0.1951 | 0.75641 |
| D_5__uncultured_delta_proteobacterium | -0.44574 | -1.4084 | 0.19667 | 0.75641 |
| D_5__uncultured_Planctomycetales_bacterium | -0.42592 | -1.3315 | 0.21972 | 0.79457 |
| D_5__bacterium_LWQ8 | -0.42363 | -1.3227 | 0.22248 | 0.79457 |
| D_5__uncultured_Acidobacteriales_bacterium | 0.39949 | 1.2326 | 0.25273 | 0.87148 |
| D_5__Gemmata | -0.37055 | -1.1284 | 0.29186 | 0.94154 |
| D_5__SH_PL14 | 0.36618 | 1.113 | 0.29802 | 0.94154 |
| D_5__Nocardioides | 0.36388 | 1.105 | 0.30129 | 0.94154 |
| D_5__Chthoniobacter | -0.34135 | -1.0272 | 0.33439 | 0.94675 |
| D_5__Pajaroellobacter | 0.32206 | 0.96218 | 0.36414 | 0.94675 |
| D_5__Pyronema_omphalodes | -0.31944 | -0.95346 | 0.36827 | 0.94675 |
| D_5__Chitinophaga | 0.30283 | 0.89872 | 0.39505 | 0.94675 |
| D_5__Defluviicoccus | 0.28336 | 0.83571 | 0.42757 | 0.94675 |
| D_5__Arenimonas | 0.27871 | 0.82085 | 0.43551 | 0.94675 |
| D_5__Candidatus_Xiphinematobacter | 0.27311 | 0.80301 | 0.44517 | 0.94675 |
| D_5__Terrimonas | 0.27179 | 0.79882 | 0.44746 | 0.94675 |
| D_5__Rubrobacter | -0.26683 | -0.78311 | 0.45612 | 0.94675 |
| D_5__uncultured_euryarchaeote | -0.24363 | -0.7105 | 0.49758 | 0.94675 |
| D_5__Subgroup_10 | 0.2402 | 0.69986 | 0.50385 | 0.94675 |
| D_5__Nitrospira | -0.23525 | -0.68459 | 0.51295 | 0.94675 |
| D_5__Rhodobacter | -0.23293 | -0.67746 | 0.51722 | 0.94675 |
| D_5__Steroidobacter | -0.23071 | -0.67062 | 0.52135 | 0.94675 |
| D_5__Adhaeribacter | 0.22053 | 0.63949 | 0.54038 | 0.94675 |
| D_5__Zavarzinella | -0.22021 | -0.63852 | 0.54098 | 0.94675 |
| D_5__uncultured_gamma_proteobacterium | -0.21852 | -0.63338 | 0.54417 | 0.94675 |
| D_5__uncultured_prokaryote | -0.21637 | -0.62685 | 0.54823 | 0.94675 |
| D_5__Pseudomonas | -0.21631 | -0.62665 | 0.54835 | 0.94675 |
| D_5__Pseudolabrys | -0.21481 | -0.62209 | 0.5512 | 0.94675 |
| D_5__uncultured_Anaerolineaceae_bacterium | -0.21296 | -0.61648 | 0.55471 | 0.94675 |
| D_5__Microseira_Carmichael_Alabama | -0.21092 | -0.6103 | 0.5586 | 0.94675 |
| D_5__OM60(NOR5)_clade | -0.1993 | -0.57524 | 0.58095 | 0.94675 |
| D_5__Gemmatimonas | 0.19694 | 0.56815 | 0.58552 | 0.94675 |
| D_5__Conexibacter | 0.19574 | 0.56455 | 0.58786 | 0.94675 |
| D_5__Peredibacter | 0.19139 | 0.55153 | 0.59634 | 0.94675 |
| D_5__RB41 | -0.19119 | -0.55092 | 0.59673 | 0.94675 |
| D_5__Altererythrobacter | 0.19064 | 0.54929 | 0.5978 | 0.94675 |
| D_5__Flavobacterium | 0.18867 | 0.54339 | 0.60167 | 0.94675 |
| D_5__Acidibacter | 0.18171 | 0.52266 | 0.61537 | 0.94675 |
| D_5__uncultured_Caldilineae_bacterium | -0.18002 | -0.51763 | 0.61872 | 0.94675 |
| D_5__Ohtaekwangia | -0.17448 | -0.50119 | 0.62973 | 0.94675 |
| D_5__Devosia | -0.17387 | -0.49939 | 0.63095 | 0.94675 |
| D_5__Ellin6067 | -0.16971 | -0.48709 | 0.63926 | 0.94675 |
| D_5__Ilumatobacter | -0.16644 | -0.47741 | 0.64584 | 0.94675 |
| Not_Assigned | -0.1651 | -0.47347 | 0.64853 | 0.94675 |
| D_5__Chlorobi_bacterium_OLB5 | -0.16265 | -0.46625 | 0.65347 | 0.94675 |
| D_5__metagenome | -0.14725 | -0.42109 | 0.68478 | 0.94675 |
| D_5__unidentified_bacterium_wb1_D18 | 0.14712 | 0.4207 | 0.68505 | 0.94675 |
| D_5__Haliscomenobacter | -0.14566 | -0.41643 | 0.68805 | 0.94675 |
| D_5__uncultured_sludge_bacterium_A31 | 0.13979 | 0.39932 | 0.70011 | 0.94675 |
| D_5__Luteolibacter | -0.13863 | -0.39593 | 0.70251 | 0.94675 |
| D_5__Sphingomonas | 0.13387 | 0.38209 | 0.71234 | 0.94675 |
| D_5__Anaeromyxobacter | 0.13041 | 0.37203 | 0.71953 | 0.94675 |
| D_5__Iamia | 0.12012 | 0.34223 | 0.74099 | 0.95444 |
| D_5__IS_44 | 0.11067 | 0.31496 | 0.76085 | 0.95444 |
| D_5__wastewater_metagenome | 0.094295 | 0.2679 | 0.79555 | 0.95444 |
| D_5__Bacillus | -0.09158 | -0.26011 | 0.80135 | 0.95444 |
| D_5__SWB02 | -0.08892 | -0.25251 | 0.80701 | 0.95444 |
| D_5__Vicinamibacter | -0.08746 | -0.24831 | 0.81015 | 0.95444 |
| D_5__OM27_clade | -0.08071 | -0.22901 | 0.8246 | 0.95444 |
| D_5__Niastella | 0.080307 | 0.22788 | 0.82546 | 0.95444 |
| D_5__Pedomicrobium | -0.07621 | -0.21617 | 0.83427 | 0.95444 |
| D_5__Lacunisphaera | 0.074875 | 0.21238 | 0.83713 | 0.95444 |
| D_5__FFCH7168 | -0.06413 | -0.18177 | 0.86029 | 0.95444 |
| D_5__Bdellovibrio | 0.062994 | 0.17853 | 0.86275 | 0.95444 |
| D_5__uncultured_subdivision_3_bacterium | 0.060523 | 0.1715 | 0.86809 | 0.95444 |
| D_5__Anaerolinea | -0.05801 | -0.16435 | 0.87353 | 0.95444 |
| D_5__Dyadobacter | -0.05791 | -0.16407 | 0.87375 | 0.95444 |
| D_5__Ramlibacter | 0.046496 | 0.13165 | 0.89851 | 0.95444 |
| D_5__Hyphomicrobium | -0.04243 | -0.12011 | 0.90736 | 0.95444 |
| D_5__Dongia | -0.03832 | -0.10845 | 0.91631 | 0.95444 |
| D_5__Pir4_lineage | 0.036333 | 0.10283 | 0.92063 | 0.95444 |
| D_5__uncultured_soil_bacterium | 0.035269 | 0.099817 | 0.92295 | 0.95444 |
| D_5__Reyranella | -0.0316 | -0.08941 | 0.93095 | 0.95444 |
| D_5__Pseudonocardia | 0.029578 | 0.083695 | 0.93536 | 0.95444 |
| D_5__uncultured | 0.025122 | 0.071079 | 0.94508 | 0.95463 |
| D_5__Bradyrhizobium | -0.02039 | -0.05768 | 0.95542 | 0.95542 |

|  | Correlation | T-Stat | P-Value | FDR |
| --- | --- | --- | --- | --- |
| D_5__Subgroup_10 | 0.79725 | 3.7355 | 0.005742 | 0.57423 |
| D_5__Defluviicoccus | 0.74663 | 3.1745 | 0.013106 | 0.65528 |
| D_5__Pseudoxanthomonas | -0.64425 | -2.3826 | 0.044367 | 0.75427 |
| D_5__uncultured_bacterium | -0.62407 | -2.259 | 0.053803 | 0.75427 |
| D_5__TG_45 | -0.62133 | -2.2429 | 0.055177 | 0.75427 |
| D_5__Nocardioides | 0.61296 | 2.1942 | 0.059526 | 0.75427 |
| D_5__Ramlibacter | 0.60997 | 2.1772 | 0.061127 | 0.75427 |
| D_5__Haliangium | -0.59631 | -2.101 | 0.068829 | 0.75427 |
| D_5__Conexibacter | 0.56294 | 1.9265 | 0.090206 | 0.75427 |
| D_5__unidentified_bacterium_wb1_D18 | 0.55995 | 1.9116 | 0.092309 | 0.75427 |
| D_5__MND1 | 0.53039 | 1.7696 | 0.11476 | 0.75427 |
| D_5__Pyronema_omphalodes | -0.52996 | -1.7676 | 0.11511 | 0.75427 |
| D_5__Desulfobulbus | -0.52792 | -1.7581 | 0.11678 | 0.75427 |
| D_5__Stenotrophobacter | -0.52381 | -1.7393 | 0.12018 | 0.75427 |
| D_5__Chryseolinea | -0.5074 | -1.6655 | 0.13439 | 0.75427 |
| D_5__Altererythrobacter | 0.50233 | 1.6432 | 0.13897 | 0.75427 |
| D_5__uncultured_bacterium_5G4 | -0.49142 | -1.5959 | 0.14917 | 0.75427 |
| D_5__Gemmata | -0.47391 | -1.5222 | 0.16646 | 0.75427 |
| D_5__uncultured_soil_bacterium | 0.46783 | 1.4972 | 0.17272 | 0.75427 |
| D_5__Pirellula | 0.45588 | 1.4487 | 0.18545 | 0.75427 |
| D_5__Gaiella | 0.43856 | 1.3803 | 0.20484 | 0.75427 |
| D_5__Bryobacter | 0.43371 | 1.3614 | 0.21048 | 0.75427 |
| D_5__Bradyrhizobium | 0.43333 | 1.36 | 0.21092 | 0.75427 |
| D_5__uncultured_sludge_bacterium_A31 | 0.42613 | 1.3323 | 0.21946 | 0.75427 |
| D_5__Anaeromyxobacter | 0.41998 | 1.3089 | 0.22691 | 0.75427 |
| D_5__uncultivated_soil_bacterium_clone_C112 | 0.41787 | 1.3009 | 0.2295 | 0.75427 |
| D_5__Candidatus_Xiphinematobacter | 0.41757 | 1.2998 | 0.22987 | 0.75427 |
| D_5__uncultured_delta_proteobacterium | -0.41523 | -1.291 | 0.23276 | 0.75427 |
| D_5__Acinetobacter | -0.41513 | -1.2906 | 0.23288 | 0.75427 |
| D_5__Pajaroellobacter | 0.41487 | 1.2896 | 0.2332 | 0.75427 |
| D_5__Iamia | 0.41437 | 1.2878 | 0.23382 | 0.75427 |
| D_5__Pir4_lineage | 0.40209 | 1.2421 | 0.24937 | 0.75511 |
| D_5__Pseudonocardia | 0.3966 | 1.222 | 0.2565 | 0.75511 |
| D_5__wastewater_metagenome | 0.3957 | 1.2187 | 0.25769 | 0.75511 |
| D_5__Dyadobacter | -0.38994 | -1.1977 | 0.2653 | 0.75511 |
| D_5__Hyphomicrobium | 0.38507 | 1.1801 | 0.27184 | 0.75511 |
| D_5__uncultured_Planctomycetales_bacterium | -0.37409 | -1.1409 | 0.2869 | 0.75763 |
| D_5__uncultured_Acidobacteriales_bacterium | 0.37337 | 1.1384 | 0.2879 | 0.75763 |
| D_5__metagenome | -0.36072 | -1.0939 | 0.30584 | 0.78419 |
| D_5__Reyranella | 0.34226 | 1.0303 | 0.33301 | 0.82027 |
| D_5__RB41 | -0.34008 | -1.0228 | 0.33631 | 0.82027 |
| D_5__IS_44 | -0.32884 | -0.98487 | 0.35353 | 0.82515 |
| D_5__Aquicella | 0.32291 | 0.96504 | 0.36279 | 0.82515 |
| D_5__mle1_7 | 0.32109 | 0.95897 | 0.36566 | 0.82515 |
| D_5__uncultured_microorganism | 0.31752 | 0.94709 | 0.37132 | 0.82515 |
| D_5__bacterium_LWQ8 | -0.30006 | -0.88969 | 0.39959 | 0.84677 |
| D_5__Niastella | -0.29893 | -0.88602 | 0.40145 | 0.84677 |
| D_5__Adhaeribacter | -0.29592 | -0.87622 | 0.40645 | 0.84677 |
| D_5__Geobacter | -0.29053 | -0.85878 | 0.41545 | 0.84785 |
| D_5__uncultured_subdivision_3_bacterium | -0.26579 | -0.77981 | 0.45795 | 0.9159 |
| D_5__Arenimonas | 0.25296 | 0.73952 | 0.48072 | 0.92811 |
| D_5__Vicinamibacter | 0.24226 | 0.70624 | 0.50009 | 0.92811 |
| D_5__uncultured_Chloroflexi_bacterium | 0.24012 | 0.69962 | 0.50399 | 0.92811 |
| Not_Assigned | -0.23663 | -0.68886 | 0.51039 | 0.92811 |
| D_5__Chitinophaga | -0.2322 | -0.67522 | 0.51857 | 0.92811 |
| D_5__FFCH7168 | 0.23157 | 0.67328 | 0.51974 | 0.92811 |
| D_5__Mycobacterium | 0.22236 | 0.64509 | 0.53693 | 0.94198 |
| D_5__Chthoniobacter | -0.19799 | -0.5713 | 0.58349 | 0.95164 |
| D_5__Lacunisphaera | 0.18268 | 0.52553 | 0.61346 | 0.95164 |
| D_5__Pedomicrobium | 0.15617 | 0.44721 | 0.66658 | 0.95164 |
| D_5__Nitrospira | -0.15297 | -0.43782 | 0.6731 | 0.95164 |
| D_5__SWB02 | 0.14753 | 0.42188 | 0.68422 | 0.95164 |
| D_5__bacterium_WX65 | 0.13041 | 0.37203 | 0.71953 | 0.95164 |
| D_5__uncultured_prokaryote | -0.12719 | -0.36271 | 0.72622 | 0.95164 |
| D_5__Devosia | 0.12477 | 0.35567 | 0.73128 | 0.95164 |
| D_5__PAUC26f | 0.11963 | 0.34081 | 0.74202 | 0.95164 |
| D_5__Dongia | 0.11304 | 0.32178 | 0.75586 | 0.95164 |
| D_5__Rubrobacter | -0.10529 | -0.29948 | 0.77221 | 0.95164 |
| D_5__Haliscomenobacter | -0.0995 | -0.28284 | 0.78448 | 0.95164 |
| D_5__Acidibacter | 0.092874 | 0.26383 | 0.79858 | 0.95164 |
| D_5__Bdellovibrio | -0.09221 | -0.26194 | 0.79999 | 0.95164 |
| D_5__Flavobacterium | -0.09206 | -0.2615 | 0.80032 | 0.95164 |
| D_5__Ilumatobacter | 0.088856 | 0.25232 | 0.80715 | 0.95164 |
| D_5__OM27_clade | -0.08664 | -0.24597 | 0.8119 | 0.95164 |
| D_5__Terrimonas | -0.08649 | -0.24556 | 0.81221 | 0.95164 |
| D_5__uncultured_gamma_proteobacterium | 0.084179 | 0.23894 | 0.81716 | 0.95164 |
| D_5__Gemmatimonas | -0.07688 | -0.21809 | 0.83282 | 0.95164 |
| D_5__SH_PL14 | -0.07335 | -0.20803 | 0.8404 | 0.95164 |
| D_5__uncultured_euryarchaeote | -0.07133 | -0.20226 | 0.84476 | 0.95164 |
| D_5__Ohtaekwangia | -0.07054 | -0.20002 | 0.84645 | 0.95164 |
| D_5__Rhodobacter | -0.0682 | -0.19333 | 0.85152 | 0.95164 |
| D_5__Steroidobacter | -0.06754 | -0.19148 | 0.85292 | 0.95164 |
| D_5__Sphingomonas | -0.06693 | -0.18974 | 0.85424 | 0.95164 |
| D_5__uncultured | 0.065085 | 0.18448 | 0.85823 | 0.95164 |
| D_5__Pseudomonas | -0.06333 | -0.17948 | 0.86202 | 0.95164 |
| D_5__uncultured_Anaerolineaceae_bacterium | -0.06235 | -0.17669 | 0.86414 | 0.95164 |
| D_5__Microseira_Carmichael_Alabama | -0.06175 | -0.17499 | 0.86543 | 0.95164 |
| D_5__Solirubrobacter | -0.06145 | -0.17412 | 0.8661 | 0.95164 |
| D_5__OM60(NOR5)_clade | -0.05835 | -0.16532 | 0.8728 | 0.95164 |
| D_5__Candidatus_Udaeobacter | -0.05762 | -0.16325 | 0.87437 | 0.95164 |
| D_5__Anaerolinea | 0.057127 | 0.16184 | 0.87544 | 0.95164 |
| D_5__Peredibacter | 0.056033 | 0.15874 | 0.87781 | 0.95164 |
| D_5__uncultured_Caldilineae_bacterium | -0.0527 | -0.14928 | 0.88503 | 0.95164 |
| D_5__Chlorobi_bacterium_OLB5 | -0.04762 | -0.13484 | 0.89607 | 0.95327 |
| D_5__Streptomyces | -0.037 | -0.10471 | 0.91918 | 0.96756 |
| D_5__Pseudolabrys | -0.02935 | -0.08305 | 0.93586 | 0.97165 |
| D_5__Ellin6067 | -0.02631 | -0.07443 | 0.9425 | 0.97165 |
| D_5__Bacillus | 0.021621 | 0.061168 | 0.95273 | 0.9718 |
| D_5__Luteolibacter | 0.013529 | 0.038269 | 0.97041 | 0.9718 |
| D_5__Zavarzinella | -0.01289 | -0.03647 | 0.9718 | 0.9718 |

|  | Correlation | T-Stat | P-Value | FDR |
| --- | --- | --- | --- | --- |
| D_5__Gaiella | 0.75931 | 3.3003 | 0.010853 | 0.62904 |
| D_5__Pirellula | 0.74944 | 3.2017 | 0.012581 | 0.62904 |
| D_5__Pseudoxanthomonas | -0.65273 | -2.4369 | 0.040759 | 0.68403 |
| D_5__MND1 | 0.61406 | 2.2006 | 0.05894 | 0.68403 |
| D_5__Acinetobacter | -0.61178 | -2.1875 | 0.060157 | 0.68403 |
| D_5__Chryseolinea | -0.60948 | -2.1744 | 0.061396 | 0.68403 |
| D_5__uncultivated_soil_bacterium_clone_C112 | 0.58502 | 2.0402 | 0.075646 | 0.68403 |
| D_5__Bryobacter | 0.58492 | 2.0397 | 0.075709 | 0.68403 |
| D_5__Defluviicoccus | 0.58071 | 2.0176 | 0.078355 | 0.68403 |
| D_5__Nocardioides | 0.57281 | 1.9766 | 0.083491 | 0.68403 |
| D_5__uncultured_microorganism | 0.53701 | 1.8006 | 0.10946 | 0.68403 |
| D_5__uncultured_bacterium | -0.52539 | -1.7465 | 0.11887 | 0.68403 |
| D_5__uncultured_euryarchaeote | -0.49929 | -1.6299 | 0.14177 | 0.68403 |
| D_5__Haliscomenobacter | -0.49752 | -1.6222 | 0.14341 | 0.68403 |
| D_5__mle1_7 | 0.49657 | 1.6181 | 0.1443 | 0.68403 |
| D_5__Aquicella | 0.49619 | 1.6164 | 0.14466 | 0.68403 |
| D_5__uncultured_Chloroflexi_bacterium | 0.49545 | 1.6133 | 0.14535 | 0.68403 |
| D_5__Mycobacterium | 0.49154 | 1.5965 | 0.14905 | 0.68403 |
| D_5__Rhodobacter | -0.47736 | -1.5366 | 0.16295 | 0.68403 |
| D_5__Steroidobacter | -0.47281 | -1.5176 | 0.16758 | 0.68403 |
| D_5__Haliangium | -0.47085 | -1.5096 | 0.16959 | 0.68403 |
| D_5__uncultured_Acidobacteriales_bacterium | 0.45659 | 1.4516 | 0.18467 | 0.68403 |
| D_5__bacterium_WX65 | 0.45644 | 1.451 | 0.18485 | 0.68403 |
| D_5__PAUC26f | 0.45091 | 1.4289 | 0.1909 | 0.68403 |
| D_5__Pseudomonas | -0.4433 | -1.3988 | 0.19943 | 0.68403 |
| D_5__Nitrospira | -0.44151 | -1.3918 | 0.20146 | 0.68403 |
| D_5__uncultured_Anaerolineaceae_bacterium | -0.43644 | -1.372 | 0.2073 | 0.68403 |
| D_5__Microseira_Carmichael_Alabama | -0.43226 | -1.3558 | 0.21219 | 0.68403 |
| D_5__Ohtaekwangia | -0.42569 | -1.3306 | 0.21999 | 0.68403 |
| D_5__Candidatus_Xiphinematobacter | 0.40868 | 1.2665 | 0.24095 | 0.68403 |
| D_5__OM60(NOR5)_clade | -0.40844 | -1.2656 | 0.24127 | 0.68403 |
| D_5__Conexibacter | 0.40114 | 1.2386 | 0.2506 | 0.68403 |
| D_5__uncultured_gamma_proteobacterium | -0.40069 | -1.237 | 0.25117 | 0.68403 |
| D_5__TG_45 | -0.39539 | -1.2176 | 0.25808 | 0.68403 |
| D_5__uncultured_soil_bacterium | 0.39476 | 1.2152 | 0.25892 | 0.68403 |
| D_5__Altererythrobacter | 0.3907 | 1.2005 | 0.26429 | 0.68403 |
| D_5__OM27_clade | -0.38592 | -1.1832 | 0.27069 | 0.68403 |
| D_5__Subgroup_10 | 0.38164 | 1.1678 | 0.27651 | 0.68403 |
| D_5__Luteolibacter | -0.37881 | -1.1577 | 0.28037 | 0.68403 |
| D_5__Pajaroellobacter | 0.37401 | 1.1406 | 0.28701 | 0.68403 |
| D_5__uncultured_prokaryote | -0.3736 | -1.1392 | 0.28758 | 0.68403 |
| D_5__Chthoniobacter | -0.36957 | -1.125 | 0.29322 | 0.68403 |
| D_5__uncultured_Caldilineae_bacterium | -0.36893 | -1.1227 | 0.29413 | 0.68403 |
| D_5__Devosia | -0.35633 | -1.0787 | 0.31219 | 0.69774 |
| D_5__Streptomyces | 0.34251 | 1.0311 | 0.33264 | 0.69774 |
| D_5__Ilumatobacter | -0.34109 | -1.0263 | 0.33478 | 0.69774 |
| D_5__Pseudonocardia | 0.33945 | 1.0207 | 0.33726 | 0.69774 |
| D_5__Chlorobi_bacterium_OLB5 | -0.33333 | -1 | 0.34659 | 0.69774 |
| D_5__Stenotrophobacter | -0.33333 | -1 | 0.34659 | 0.69774 |
| D_5__Geobacter | -0.32979 | -0.98806 | 0.35206 | 0.69774 |
| D_5__Ellin6067 | -0.32735 | -0.97987 | 0.35585 | 0.69774 |
| D_5__Desulfobulbus | -0.31415 | -0.93593 | 0.3767 | 0.72442 |
| D_5__uncultured_bacterium_5G4 | -0.30771 | -0.91472 | 0.38708 | 0.73033 |
| D_5__unidentified_bacterium_wb1_D18 | 0.30151 | 0.89443 | 0.3972 | 0.73556 |
| D_5__Solirubrobacter | 0.2957 | 0.87553 | 0.4068 | 0.73964 |
| D_5__uncultured_sludge_bacterium_A31 | 0.28649 | 0.84578 | 0.42225 | 0.75402 |
| D_5__Anaeromyxobacter | 0.26726 | 0.78446 | 0.45537 | 0.79417 |
| D_5__Candidatus_Udaeobacter | 0.26427 | 0.77503 | 0.46062 | 0.79417 |
| D_5__Bdellovibrio | -0.2582 | -0.75593 | 0.47136 | 0.79892 |
| D_5__uncultured_delta_proteobacterium | -0.24914 | -0.72761 | 0.4876 | 0.80812 |
| D_5__Iamia | 0.24618 | 0.7184 | 0.49296 | 0.80812 |
| D_5__Gemmata | -0.23981 | -0.69867 | 0.50456 | 0.81381 |
| D_5__Pir4_lineage | 0.22338 | 0.6482 | 0.53501 | 0.82536 |
| D_5__FFCH7168 | 0.21905 | 0.635 | 0.54316 | 0.82536 |
| D_5__Pyronema_omphalodes | -0.21822 | -0.63246 | 0.54474 | 0.82536 |
| D_5__uncultured_Planctomycetales_bacterium | -0.21822 | -0.63246 | 0.54474 | 0.82536 |
| D_5__Chitinophaga | 0.20687 | 0.59805 | 0.56635 | 0.83026 |
| D_5__Anaerolinea | -0.20535 | -0.59346 | 0.56927 | 0.83026 |
| D_5__Dongia | -0.19933 | -0.57533 | 0.58089 | 0.83026 |
| D_5__bacterium_LWQ8 | -0.19604 | -0.56545 | 0.58727 | 0.83026 |
| D_5__wastewater_metagenome | 0.19325 | 0.55709 | 0.59271 | 0.83026 |
| D_5__Bacillus | 0.18767 | 0.54042 | 0.60362 | 0.83026 |
| D_5__SWB02 | -0.18224 | -0.52422 | 0.61433 | 0.83026 |
| D_5__Vicinamibacter | -0.17923 | -0.51528 | 0.62029 | 0.83026 |
| D_5__Dyadobacter | -0.17802 | -0.51168 | 0.62269 | 0.83026 |
| D_5__uncultured | -0.1564 | -0.44787 | 0.66612 | 0.86569 |
| D_5__Pedomicrobium | -0.15617 | -0.44721 | 0.66658 | 0.86569 |
| D_5__Adhaeribacter | 0.15065 | 0.43102 | 0.67783 | 0.86902 |
| D_5__Sphingomonas | -0.13085 | -0.3733 | 0.71862 | 0.89924 |
| D_5__Flavobacterium | -0.12888 | -0.36761 | 0.7227 | 0.89924 |
| D_5__uncultured_subdivision_3_bacterium | 0.12403 | 0.35355 | 0.73281 | 0.89924 |
| D_5__SH_PL14 | 0.11849 | 0.33753 | 0.74441 | 0.89924 |
| D_5__Niastella | -0.11756 | -0.33482 | 0.74637 | 0.89924 |
| D_5__Lacunisphaera | -0.1023 | -0.29087 | 0.77855 | 0.92684 |
| D_5__Ramlibacter | 0.095288 | 0.27075 | 0.79344 | 0.93346 |
| D_5__Arenimonas | 0.085679 | 0.24323 | 0.81395 | 0.94645 |
| D_5__Gemmatimonas | -0.06727 | -0.19069 | 0.85352 | 0.97043 |
| Not_Assigned | -0.06658 | -0.18874 | 0.85499 | 0.97043 |
| D_5__RB41 | -0.06256 | -0.17729 | 0.86369 | 0.97043 |
| D_5__Zavarzinella | 0.045129 | 0.12778 | 0.90148 | 0.97734 |
| D_5__Bradyrhizobium | -0.04178 | -0.11828 | 0.90876 | 0.97734 |
| D_5__Hyphomicrobium | -0.03727 | -0.10547 | 0.9186 | 0.97734 |
| D_5__Reyranella | 0.032376 | 0.091622 | 0.92925 | 0.97734 |
| D_5__Pseudolabrys | 0.029348 | 0.083045 | 0.93586 | 0.97734 |
| D_5__Terrimonas | -0.02422 | -0.06852 | 0.94705 | 0.97734 |
| D_5__IS_44 | -0.02413 | -0.06827 | 0.94725 | 0.97734 |
| D_5__Rubrobacter | -0.02378 | -0.06727 | 0.94802 | 0.97734 |
| D_5__metagenome | 0.0191 | 0.054033 | 0.95823 | 0.97779 |
| D_5__Acidibacter | 0.006312 | 0.017853 | 0.98619 | 0.99615 |
| D_5__Peredibacter | -2.41E-17 | -6.81E-17 | 1 | 1 |
